# Manipulation of plant height in garden asparagus (*Asparagus officinalis* L.) through CRISPR/Cas9-mediated *aspSPL14* allele editing

**DOI:** 10.1093/hr/uhad096

**Published:** 2023-05-10

**Authors:** Jingsong Zhou, Reqing He, Xiaojing Liu, Bingbing Zhang, Guangyu Chen, Dong Wang, Youlin Zhu

**Affiliations:** Key Laboratory of Molecular Biology and Gene Engineering in Jiangxi Province, College of Life Science, Nanchang University, Nanchang Jiangxi, 330031, China; Institute of Vegetables and Flowers, Jiangxi Academy of Agricultural Sciences, Nanchang Jiangxi, 330200, China; Key Laboratory of Molecular Biology and Gene Engineering in Jiangxi Province, College of Life Science, Nanchang University, Nanchang Jiangxi, 330031, China; Institute of Vegetables and Flowers, Jiangxi Academy of Agricultural Sciences, Nanchang Jiangxi, 330200, China; Institute of Vegetables and Flowers, Jiangxi Academy of Agricultural Sciences, Nanchang Jiangxi, 330200, China; Institute of Vegetables and Flowers, Jiangxi Academy of Agricultural Sciences, Nanchang Jiangxi, 330200, China; Key Laboratory of Molecular Biology and Gene Engineering in Jiangxi Province, College of Life Science, Nanchang University, Nanchang Jiangxi, 330031, China; Key Laboratory of Molecular Biology and Gene Engineering in Jiangxi Province, College of Life Science, Nanchang University, Nanchang Jiangxi, 330031, China

Dear Editor,

Reduction in plant height has been associated with yield increases and yield stability in a number of important crop species, such as wheat and rice [1]. In these plants, dwarfing is mainly attributed to the inability to synthesize or respond to certain phytohormones, predominantly gibberellin (GA) [2]. *Ideal Plant Architecture 1* (*IPA1*), an miR156 target gene, encodes SPL14 and it is able to bind directly to the promoters of multiple GA biosynthetic, signal, and deactivating genes in rice [3]. Moreover, *IPA1* loss-of-function mutants exhibit dwarf phenotypes [4].

Garden asparagus (*Asparagus officinalis* L.), known as the ‘king of vegetables’, is one of the top 10 most popular vegetables owing to its unique texture, taste, and high nutritional value. This species is a diecious and perennial plant with a 1C genome size of 1.3 Gb and 2*n* = 2*x* = 20 chromosomes [5, 6]. Despite its agronomic importance, it is difficult and time-consuming to modify asparagus traits by traditional breeding due to the diecious nature and the narrow genetic base of the diploid cultivars. The application of biotechnology in asparagus breeding has been lagging due to the lack of tools for efficient genetic manipulation.

The recently characterized clustered regularly interspaced short palindromic repeat (CRISPR)-associated protein 9 (Cas9) has been successfully applied to induce site-specific double-strand DNA breaks (DSBs) for genome editing in numerous plant species [7], but it has never been applied to asparagus. Here, we report successful mutation of the *SPL14* allele to produce dwarf asparagus plants using the CRISPR/Cas9 system.

To identify the gene homologous to *IPA1* from garden asparagus, a maximum likelihood tree was constructed using 17 SPL asparagus proteins and the rice IPA1 protein with MEGA5 software. The phylogenetic tree showed that AsparagusV1 05.1067 was more closely related the rice IPA1 protein than other SPL asparagus proteins, indicating that AsparagusV1 05.1067 is an *IPA1* homolog from garden asparagus (Fig. 1A). Therefore, we named it *aspSPL14* in this study. To examine the role of *aspSPL14* in the regulation of plant height, we developed a targeted mutation system dependent on CRISPR/SpCas9 in garden asparagus. The *35S* promoter-driven SpCas9 was fused to a 3 × FLAG tag and appropriate nuclear localization signals (NLSs), and then an *A. officinalis* promoter was cloned and applied to express sgRNA, leading to the vector pAspU6-SpCas9 (Fig. 1B).

**Figure 1:**
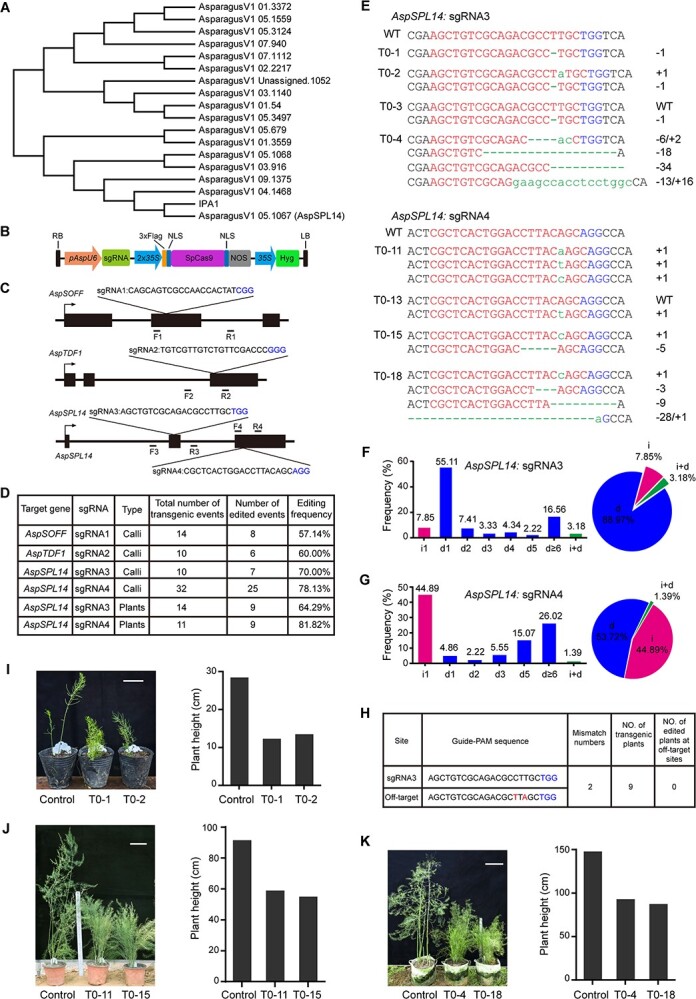
Targeted mutagenesis of *SPL14* by using pAspU6-SpCas9 in garden asparagus. (**A**) A phylogenetic tree was constructed using the full-length SPL protein sequences from garden asparagus and rice IPA1 in MEGA5. (**B**) Schematic view of pAspU6-SpCas9. (**C**) Schematic view of structures of *AspSOFF*, *AspTDF1*, and *AspSPL14* loci. The target sites and primers used are shown. Right angle arrow shows the direction of gene transcription. Boxes and lines indicate exons and introns, respectively. The four primer pairs shown (F1 and R1, F2 and R2, F3 and R3, F4 and R4 for the sgRNA1 target site, sgRNA2 target site, sgRNA3 target site and sgRNA4 target site, respectively) were designed to amplify specific target regions. (**D**) Statistics of edited garden asparagus calli and plants at target sites of different sgRNAs. The editing frequency was calculated by dividing the number of editing events by the total number of transgenic events. (**E**) Representative *AspSPL14* mutations at the sgRNA3 and sgRNA4 sites. SgRNA target regions are highlighted in red. Green lowercase letters are used to indicate indels. Deleted nucleotides are represented by green lines. PAM regions are shown in blue. (**F**) Mutation types (left) and indel frequencies (right) at the sgRNA3 site of *AspSPL14*. Letters ‘i’ and ‘d’ stand for bp of insertion or deletion, respectively. (**G**) Mutation types (left) and indel frequencies (right) at the sgRNA4 site of *AspSPL14*. (**H**) Statistics of edited transgenic asparagus plants at potential off-target site of sgRNA3. (**I**–**K**) Phenotypes of transgenic control and the *spl14* mutant plants. (**I**) Growth phenotypes (left) and plant heights (right) of 3-month-old transgenic control, *T*_0–1_ and *T*_0–2_ plants. Scale bar: 5 cm. Transgenic control plants were transformed with the vector pAspU6-SpCas9 but no sgRNA. (**J**) Growth phenotypes (left) and plant heights (right) of 8-month-old transgenic control, *T*_0–11_ and *T*_0–15_ plants. Scale bar, 10 cm. (**K**) Growth phenotypes (left) and plant heights (right) of 18-month-old transgenic control, *T*_0–4_ and *T*_0–18_ plants. Scale bar, 20 cm.

To determine the ability of SpCas9 to cause mutation in the garden asparagus genome, the editing frequency of SpCas9 in calli was first examined. Three garden asparagus endogenous genes, *aspSOFF*, *aspTDF1*, and *aspSPL14*, were selected as target genes, and four independent sgRNAs were designed to target them (Fig. 1C). A total of 66 transgenic calli were generated by *Agrobacterium*-mediated transformation, and the target sites were amplified and analyzed using PCR and Sanger sequencing. We found SpCas9-induced editing frequencies ranging from 57.14 to 78.13% across the four target loci (Fig. 1D, Supplementary Data Figs S1– S4), which indicates that pAspU6-SpCas9 successfully produces endogenous gene editing in garden asparagus.

To further validate the contribution of *aspSPL14* to dwarfing in garden asparagus, 18 resistant shoots from transgenic calli, including sgRNA3 and sgRNA4, were recovered for the following analysis (Fig. 1D). By sequencing, we found that two *T*_0_ lines separately exhibited homozygous (11.1%) and biallelic (11.1%) mutations at the sgRNA3 target site, while seven of the *T*_0_ lines showed heterozygous (one out of nine) or chimeric (six out of nine) mutations (Fig. 1E, Supplementary Data Figs S5 and S7). The most frequent indel (insertion and deletion) type was deletion (88.97%) at this target site, of which 55.11% were deletions of 1 bp (Fig. 1F). For the mutation efficiency at the sgRNA4 target site, none of the homozygous mutations was observed and only one biallelic (11.1%) mutation was found in nine of the *T*_0_ lines, but eight of the *T*_0_ lines presented heterozygous (one out of nine) or chimeric (seven out of nine) mutations (Fig. 1E, Supplementary Data Figs S6 and S8). The most frequent indel (insertion and deletion) type was insertion of 1 bp (44.89%) at the sgRNA4 target site (Fig. 1G). Besides introducing indels in the garden asparagus genome, SpCas9 could induce combined indel frequencies of 3.18% (sgRNA3) and 1.39% (sgRNA4) (Fig. 1F and G). The specificity of SpCas9 was also evaluated in garden asparagus. Potential off-target sites of both sgRNA3 and sgRNA4 were analyzed using the online tool CRISPR-GE [8], suggesting that a potential off-target site for sgRNA3 had two mismatches and the potential off-target site for sgRNA4 contained more than five mismatches. Therefore, the potential off-target site for sgRNA3, AsparagusV1 03.1140 (*AspSPL1*), was chosen for further experiments and sequencing, which did not reveal any editing events (Fig. 1H). Taken together, ~89% of deletions were produced by pAsp6-SpCas9 at sgRNA3 target sites, and pAsp6-SpCas9 induced nearly half of the insertions at the sgRNA4 target site.

Next, phenotypic development of plant height was examined in two *T*_0_ lines harboring mutations at the sgRNA3 target site: *T*_0–1_, with a homozygous mutation, and *T*_0–2_, with a biallelic mutation, exhibited evidently reduced height compared with the transgenic control plant (Fig. 1I). Moreover, an obvious decrease in plant height was observed in both *T*_0–11_ and *T*_0–15_, which held chimeric mutations at the sgRNA4 target site (Fig. 1J). Two *T*_0_ lines, *T*_0–4_, with a chimeric mutation at the sgRNA3 target site, and *T*_0–18_, harboring a chimeric mutation at the sgRNA4 target site, also presented remarkably decreased height compared with the control (Fig. 1K). These results together prove that *aspSPL14* mutation induced by pAspU6-SpCas9 causes a decrease in plant height in garden asparagus.

In summary, we successfully carried out site-specific manipulation of garden asparagus genes using the CRISPR/Cas9 system. In addition, loss of *aspSPL14* in garden asparagus was shown to generate mutants with reduced plant height. Tip pruning is a normal farming practice to prevent lodging in asparagus production in China. Considering that semi-dwarf plant architecture can improve lodging resistance of garden asparagus plants, it will be very useful to generate semi-dwarf garden asparagus lines in asparagus breeding. In addition, mutation of *IPA1* could affect plant architecture, such as plant height, tiller number, and flower number [4, 9]. Indeed, an increased number of shoots along with plant height reduction was observed in edited transgenic asparagus plants (Fig. 1J and K), and a comprehensive study regarding the effect of *aspSPL14* on asparagus shoot development will be performed in the future. More importantly, ~89% of transgenic plants exhibited no wild-type genome, and clearance of the wild-type genome in the first generation of transgenic lines will be useful for garden asparagus because this species can be propagated *in vitro*. Our findings demonstrate the applicability of CRISPR/Cas9 in garden asparagus, thereby facilitating both the genetics and the breeding of this important crop species in future.

## Acknowledgements

This work was funded by the National Natural Science Foundation of China (grants 31960433 and 31860562) and Natural Science Foundation of Jiangxi Province (grant 20171ACB20001).

## Author contributions

Y.Z. and D.W. conceived the project. D.W. and R.H. designed the experiments and wrote the manuscript. J.Z., X.L., and B.Z. performed the experiments. J.Z., R.H., D.W., Y.Z., and G.C. analyzed the results. All authors read and approved the final manuscript.

## Data availability

Data generated or analyzed during this study are included in this article and its supplementary information files.

## Conflict of interest

The authors declare no conflict of interest.

## Supplementary Data

Supplementary data is available at *Horticulture Research* online.

## Supplementary Material

Web_Material_uhad093
